# “Snake-oil,” “quack medicine,” and “industrially cultured organisms:” biovalue and the commercialization of human microbiome research

**DOI:** 10.1186/1472-6939-13-28

**Published:** 2012-10-30

**Authors:** Melody J Slashinski, Sheryl A McCurdy, Laura S Achenbaum, Simon N Whitney, Amy L McGuire

**Affiliations:** 1 Center for Medical Ethics and Health Policy, Baylor College of Medicine, One Baylor Plaza, Houston, TX 77030, USA; 2 University of Texas School of Public Health, Center for Health Promotion and Prevention Research, 7000 Fannin Street, Houston, TX, 77030, USA; 3 Department of Family and Community Medicine, Baylor College of Medicine, 3701 Kirby Drive, Suite 600, Houston, TX, 77098, USA

**Keywords:** Commercialization, Human microbiome, Ethical legal and social implications (ELSI), Dietary supplements, Qualitative research

## Abstract

**Background:**

Continued advances in human microbiome research and technologies raise a number of ethical, legal, and social challenges. These challenges are associated not only with the conduct of the research, but also with broader implications, such as the production and distribution of commercial products promising maintenance or restoration of good physical health and disease prevention. In this article, we document several ethical, legal, and social challenges associated with the commercialization of human microbiome research, focusing particularly on how this research is mobilized within economic markets for new public health uses.

**Methods:**

We conducted in-depth, semi-structured interviews (2009–2010) with 63 scientists, researchers, and National Institutes of Health project leaders (“investigators”) involved with human microbiome research. Interviews explored a range of ethical, legal, and social dimensions of human microbiome research, including investigators’ perspectives on commercialization. Using thematic content analysis, we identified and analyzed emergent themes and patterns.

**Results:**

Investigators discussed the commercialization of human microbiome research in terms of (1) commercialization, probiotics, and issues of safety, (2) public awareness of the benefits and risks of dietary supplements, and (3) regulation.

**Conclusion:**

The prevailing theme of ethical, legal, social concern focused on the need to find a balance between the marketplace, scientific research, and the public’s health. The themes we identified are intended to serve as points for discussions about the relationship between scientific research and the manufacture and distribution of over-the-counter dietary supplements in the United States.

## Background

Continued advances in human microbiome research and technologies raise a number of ethical, legal, and social challenges. These challenges are associated not only with the conduct of the research itself, such as privacy, informed consent, return of research results, invasiveness of sampling, and participant diversity [1], but also with broader implications ranging from health care services and delivery, to peoples’ conceptions of health and disease, to the mass production and distribution of commercial products promising maintenance or restoration of good physical health and disease prevention. In this article we are concerned with the latter implication. Drawing from a larger qualitative study on the ethical, legal, and social dimensions of human microbiome research, the findings we present here focus on the commercialization of human microbiome research, particularly the mobilization of this research within economic markets for new public health uses.

In the United States, the use of dietary supplements ^1^ is becoming increasingly common [2]. According to the Institute of Medicine [3], the dietary supplements industry is one of the fastest growing industries, developing an annual average of 1,000 new products, and netting billions of dollars in annual revenue. Dietary supplements, including over-the-counter probiotic-containing food products such as yogurt, cereal, and wellness bars, are overwhelmingly marketed to a generally healthy population [4] under the umbrella of *functional foods*, or “whole foods and fortified, enriched, or enhanced foods [that] have a potentially beneficial effect on health when consumed as part of a varied diet on a regular basis, at effective levels” [ [5] p.735]. Marketing strategies promote these added health benefits in terms of “vitality,” “balance,” and “beauty,” and claim relief from digestive irregularity to acne. Recent research on public perceptions of these products suggests these strategies are lucrative, as products are becoming essential to peoples’ preventative health care regimens [2], and consumers generally perceive these products as both safe and effective [6].

The growth of this industry introduces several ethical, legal and social concerns about the commercialization of human microbiome research. Notably, how dietary supplements are mobilized within economic markets and gain social currency for new public health uses, often without evidence to substantiate safety or effectiveness [7], and with sparing regulation or oversight. We contend that the relationship between scientific research and commercialization manifests in terms of both a perceived public value and a marketable exchange value, or what sociologist Catherine Waldby [8] refers to as *biovalue*. To illustrate, we consider the commercial potential of large-scale human microbiome research efforts, particularly the Human Microbiome Project, that identify human microbial communities and *good* bacteria as having “biological vitality” [ [8] p. 310]. The public value centers on expectations that these research efforts will provide a “viable contribution to human health” [ [8] p. 310], while the marketable exchange value centers on expectations that industry will profit substantially from natural or engineered biological commodities under the guise of contributing to human health. To date, there is a dearth of research as to how the commercialization of human microbiome research might further compound the ethical, legal, and social issues debated in the context of maintenance or restoration of good physical health and the prevention of disease or illness. Our purpose here is to begin to explore and discuss some of these issues, and their broader implications.

### The Human Microbiome Project

The Human Microbiome Project (HMP), initiated in 2007 by the National Institutes of Health (NIH), aims to characterize the role human microbiota, or the collections of bacteria, viruses, and other microorganisms that inhabit human bodies, play in human health and disease. The HMP seeks to establish a relationship between microbial communities and human health, human behavior, and the environment, investigating whether or not individuals share a core human microbiome, and whether or not changes in the human microbiome are correlated with changes in human health [9]. This large-scale, multi-site research effort was intended to yield not only clinical [10], pharmaceutical [11], and other public health [12] benefits, but also a reference set of microbial genome sequences, a repository to store biological materials, and new technologies, including tools for computational analysis, interpretation, and the Data Analysis and Coordinating Center (DACC) [13]. Since its inception, researchers studying the human microbiome have associated the presence or absence of certain good bacteria with a number of chronic and acute conditions including atherosclerosis [14], inflammatory bowel disease [15], colon cancer [16], irritable bowel syndrome [17], nonalcoholic fatty liver disease [18], and perinatal and neonatal health [19]. Additionally, bioinformaticists have developed specific tools and techniques to analyze, interpret, and store these data [20-22].

## Methods

Drawing from a larger qualitative study exploring the ethical, legal, and social dimensions of human microbiome research, the findings presented here emerged from 60 semi-structured interviews we conducted with 63 scientists, researchers, and NIH project leaders (“investigators”) engaged in human microbiome research (2009–2010). Semi-structured interviews allowed us to set the agenda in terms of the topics we wanted to cover, while providing investigators flexibility in their discussions and interpretations of these topics. These semi-structured interviews lasted between 30 and 90 minutes, and consisted of a series of open-ended questions exploring ethical, legal, and social challenges associated with human microbiome research. Topics discussed included investigator role; ethical challenges; clinical applications and future ethical clinical implications; precedents for managing ethical, legal, and social issues; ethical, legal, and social issues unique to microbiome research; and other topics as they arose see Additional file 1. In order to achieve a diverse sample of clinical, applied, and research expertise, we employed two sampling strategies. Initially, using a purposive sampling strategy [23], we identified 140 potential investigators from a publicly accessible list of HMP Research Network Meeting Participants. Subsequent snowball sampling [23] supplemented our recruitment efforts, allowing us to interview investigators who, for example, were not directly involved in the HMP but were involved in human microbiome research. Eighty-eight agreed to participate, 41 did not respond, and 11 declined. Of the 88 investigators who agreed to participate, 63 were interviewed in 60 distinct interviews (the remaining 25 were not interviewed due to scheduling conflicts). Investigators represented a range of academic disciplines, including genetics, pathology, microbiology, virology, gastroenterology, and medicine.

All interviews were conducted in-person in the Baylor College of Medicine Center for Medical Ethics and Health Policy conference room, in investigators’ offices, or during the January 2010 and August 2010 Human Microbiome Consortium meetings (Houston, Texas and St. Louis, Missouri, respectively). Interviews were digitally recorded and transcribed verbatim by an independent transcriptionist service. We analyzed data (i.e. interview transcripts) using thematic content analysis. Two authors (MJS, LSA) independently coded transcripts and reached consensus in coding [24] using ATLAS.ti (v 6.2), a qualitative data analysis software program. ATLAS.ti files were distributed to other members of the research team for additional comment and coding. To protect confidentiality, we refer to investigators using an identification number (Investigator #), given in parentheses after quotations or references attributable to them. Investigators provided verbal consent, and all procedures were reviewed and approved by the Baylor College of Medicine Institutional Review Board and the University of Texas Health Science Center Committee for the Protection of Human Subjects. A comprehensive explanation of our methods has been previously documented [25].

We limit our findings to the ethical, legal, and social considerations raised by human microbiome research that intersect with commercialization related issues, such as safety, risks and benefits, public awareness, and regulation. Below, we present each of these issues, paying particular attention to the ethical, legal, and social aspects of commercialization and human microbiome research. We conclude considering broader implications for public health and recommendations for policy change.

## Results and Discussion

### Commercialization, probiotics, and issues of safety

Human microbiome research is expected to provide a valuable contribution to human health and disease prevention. One area of interest centers on investigating the utility of good bacteria, often delivered through prebiotic, probiotic, or promicrobial therapies and treatments [26], for a range of acute and chronic diseases (i.e. diseases of the gastrointestinal tract). These and similar investigations bolster the scientific value of good bacteria, opening a door for “claims made by commercial organizations [that] cite the Human Microbiome Project and all of its findings as evidence that customers should buy their product.” (Investigators #154) Investigators described the ways in which these claims have the potential to manifest in widespread commercialization of a range of over-the-counter probiotics, for example:

"I also see a huge push for probiotic anything. We always joke about probiotic lotion, probiotic chewing gum—I think they’ll try to work probiotics into anything and everything they can. There’s data out there that shows these communities are important. They’re important in everything—from helping your immune system get started to breaking down the food that you eat, to just producing vitamins. So I really think that probiotics will be huge. There’ll be many, many more, and it will be for the craziest things but—like, you think of probiotic shampoo. I bet they can market probiotic shampoo to cure dandruff or something. It will be something crazy. Jergens will come out with probiotic lotion, and Extra chewing gum. (Investigator #135)"

Investigators recognized the currency of these commercial products, thus they were attuned to the ways in which scientific value is mobilized within markets to yield a public value, namely improving health through the use of these products. There were those who expressed concerns with this process, offering their insights into the myriad of problems associated with this burgeoning industry, specifically issues related to unsubstantiated health claims and safety. One investigator described the safety issues associated with products that emphasize natural ingredients while minimizing synthetic or bioengineered ingredients:

"There’s a bazillion packets for probiotics out there now. None of them have been validated. They all have organisms that are proprietary, you don’t know what’s in the box…that you’re being fed industrially cultured organisms that at one point in their lives were probably related to something natural, but over the many, many zillions of generations they’ve been fermented. They’re no longer a wild organism, they are an industrial organism. And we eat them and they may be replacing normal flora. We live in a highly processed environment and it seems that most studies are showing that experiencing a diverse collection of wild organisms is what we evolved from and what helped train our immune system. We—because we’re more industrialized, we’re probably experiencing less exposure to organisms. Organisms live in environments. Urban environments may be less rich in diversity and they may be less rich in the diversity we need. We sterilize all our vegetables, irradiate it, and we grow our animals in mass culture. We propagate huge outbreaks of Staph Aureus and infectious organisms that are now antibiotic resistant. All these are now becoming the organisms we’re introduced to—monocultures of organisms…but we are what we eat and we are eating things that are industrialized. (Investigator #133)"

There were investigators who described the commercialization of dietary supplements, including probiotics, in terms of “fad[s]” (Investigator #119) or “quack medicine” (Investigator #104) not only because the claims made on behalf of these over-the-counter products are unsubstantiated, but also because, like the products, the health claims are manufactured for the explicit purpose of turning a profit as opposed to actually improving the health of the consumer. One investigator described placing the interests of the market over the interests of consumers, as “I’ll bet you this is a problem we only think about in Western capitalistic societies where we’re worried about profit as opposed to community knowledge and community well-being.” (Investigator #111)

Scientific debates over the effectiveness and safety of dietary supplements seem to have had little influence on their popularity as health products. Even though there is a dearth of scientific evidence to support the health benefits conveyed by these products [27,28], the reputed health benefits might be the motivating factor that underscores the continued use of these products. The investigators with whom we spoke were aware of the growing popularity of these products, and while concerned, they were not surprised that “people still keep using them.” (Investigator #133) In one sense, continued use of these products may be a testament to the power of commercialization in a health oriented consumer culture. Nettleton [29] refers to this culture as a *new paradigm of health*, which emphasizes individual responsibility and lifestyle changes for ensuring good health and preventing disease. Marketing strategies adopt this rhetoric of individual responsibility, constructing the body in a state of imbalance, providing consumers with a choice to be healthy, and then encouraging them to be proactive. Within this new paradigm of health, marketing strategies do not need to include an explanation of why products are good for health, they only need to present products as simple or easy ways in which individuals can take responsibility for their health: “[p]eople just get excited because they think it’s going to magically solve their health problems [and] assume that something’s going to deliver more than it says, than it really can.” (Investigator #119)

### Public awareness of the benefits and risks of dietary supplements

Investigators expressed strong opinions about the ways in which the dietary supplements industry exploits and manipulates scientific research to secure a profit, largely at the expense of the public:

"I think it’s an industry that preys on people that will look for alternative forms of medicine – which I don’t think are bad…I totally believe in holistic medicine…but there are some people who, because of the ability of advertising for those kinds of products, can be swayed to use and spend their money for things that are probably of no value. (Investigator #128)"

Commercial entities not only take advantage of scientific research, but also of an unaware public striving to achieve healthy bodies. Sharp and colleagues [7] reviewed several frameworks concerning public awareness regarding the benefits and risks of dietary supplements. They introduced several safety-related considerations, specifically in the use of probiotics, including the unpredictable behavior of both naturally occurring and genetically altered microorganisms, each of which have the potential to produce substances or gene-behaviors that are harmful to the body. Investigators contend, however, that the allure of good health trumps the risks, and results in these products being consumed more as an article of faith [27]:

"I think the biggest [issue] is just making sure that people are really aware of what they’re doing. As an example, if probiotics do become the big thing, just making sure that people understand what they’re taking and understand that it’s not just some magical cure all, and that there are risks involved, also…[I]f people are thinking, ‘Oh, probiotics are just a yogurt,’ or something easy like that…you don’t want them to get into that mindset that ‘Oh this is something that’s all good and there aren’t any risks associated.’ I don’t know of any risks associated with eating Activia, but they just need to be aware that probiotics can do negative things, as well – can alter your microbiome in a bad way. (Investigator #156)"

Investigators were asked to share their thoughts about the most effective ways to increase the public’s awareness about the risks and benefits of dietary supplements. Many focused on the need to use productive and understandable language that serves to inform, rather than inflame or scare, consumers:

"I think interfacing with the popular media is gonna be really key there. And that’s so difficult, because there’s a temptation to make – you know, put out speculative headlines based on the first announcement of some new disease association and those things can stick in the public’s mind in a way that’s not productive. (Investigator #145)"

However, investigators were aware that translating and disseminating this information has to be done in such a way that the general or lay public will understand. This was discussed in terms of the benefits and risks of including these products in a daily diet, for example the health implications of changing the balance between different bacterial species, as well as understanding what this information means for them individually.

### Regulation

In October 1994, the Dietary Supplement Health and Education Act (DSHEA) was signed into law, amending the federal Food, Drug, and Cosmetic Act [30]. DSHEA loosened restrictions on developers of dietary supplements by requiring only that the “representations or claims [are] substantiated by adequate evidence to show that they are not false or misleading.” This means that dietary supplements are not subject to approval from the US Food and Drug Administration (FDA) prior to marketing and distribution, nor are developers required to provide the FDA with evidence to substantiate safety or effectiveness before or after it markets its products. DSHEA places dietary supplements in a special category under the general umbrella of foods, not drugs, thus only requires that every supplement be labeled a dietary supplement. The lack of regulation was perceived as a political move purposed to bolster the economic potential of the dietary supplements industry:

"The probiotics industry is not accustomed to being regulated. There will be political agitation against it. It will probably be a bit like the vitamin supplements industry…maybe 10 or 15 years ago [the] FDA attempted to start regulating those, and there was furious backlash from the public. Congress passed a law, which actually prevented [the] FDA from regulating these supplements…The backlash was both from the public, because it turns out millions and millions of people take these things, and they don’t want their access restricted, of course. It’s big business, and of course big business doesn’t want to be regulated, and so there was a backlash just on all fronts against that. (Investigator #113)"

Investigators perceived the politicization of these issues as harmful to the public’s health:

"[P]eople can sell you snake-oil treatments to deal with your bad bacteria…I think if people sell stuff and they say ‘I’m selling you,’ I don’t know, ‘cypress leaves’ I think you should have some guarantee that in fact there’s cypress leaves in there, not arsenic. And just because it’s sold by a health food store or it’s a medical food or it’s some other crap, you should have some assurance that it’s not going to hurt you, and what’s really in there is in there. And that should be across the board. (Investigator #108)"

An oversight committee, ideally one that does not have a vested economic interest and is charged primarily with protecting public health, should validate product assertions. Investigators recognized that disclosing product ingredients is important, and measures requiring full disclosure would prohibit the industry from misleading assertions. Overall, their concerns were less about use of these products, and more about how lack of regulation has the potential to negatively affect health. This could result in issues of where to draw the line:

"So, the science that I’m in, you can see sort of ethical implications of it when you walk through the drug store and you look at the homeopathic medicines and you see claims that are meant to look [like] scientific claims that are not supported by science. And, it turns out, that if it were a medicine, it would be regulated in one way—that you can’t claim that the medicine helps unless it does, because it’s medicine. But anything that’s a food, you can make a different set of claims without the need for validation of these claims. The Human Microbiome Project ends up being a whole lot of science about bacteria growing in the gut as the body site that most closely ties to what we eat, and the connection between what we eat and health is potentially exploited by untold numbers of people in the sort of quack science of homeopathic medicine – the weak to nonexistent science of most probiotics – the claims that every breakfast cereal company wants to make. (Investigator #155)"

Taken together, investigators’ insights into regulatory issues centered on the intersection of the public value and a marketable exchange value, and the implications of this to create more harm than good. Dietary supplements assert unsubstantiated health claims, and the current lack of regulation ensures the dietary supplements industry will continue to profit from these unsubstantiated claims. These issues are directly related to the public’s health and wellbeing, and investigators not only recognized these issues but, overall, support stricter regulations that place the public’s health over industry profits.

## Conclusion

As previously stated, there are a number of ethical, legal and social issues associated with human microbiome research. In this article, we focused primarily on those issues concerning the commercialization of human microbiome research. In our examination we have considered how human microbiome research has biovalue, illustrating the power of the dietary supplements industry, and the need to find a balance between the marketplace, scientific research, and the public’s health. Investigators’ responses are less about the authority or expertise of biomedicine, and more about how biomedical research is co-opted by commercial entities that place profit over health. Investigators focused on the power of the industry, namely it is not about the products themselves, but what the public is required to know about the products. For example, a product that claims “all natural ingredients” should be natural, not engineered in a lab. The themes we identified are intended to serve as points for discussions about the relationship between scientific research and the manufacture and distribution of over-the-counter dietary supplements.

Spielmans and Parry [31] note that prior to understanding the utility of scientific data “science has largely been taken captive in the name of increasing profits” (p. 13). Investigators’ responses suggest the commercialization of human microbiome research, for example the production and distribution of over-the-counter dietary supplements, is predicated on mass consumption, not health or wellbeing, in pursuit of potential profits. They suggest human microbiome research opens the door for what we will refer to as a *commercialized intervention*, or the proliferation of commercial products that claim maintenance or restoration of *good* health, and prevention of disease or sickness, with the use of *good* bacteria. Under the current paradigm of health that encourages individual responsibility, we need to consider why individuals begin or continue to use dietary supplements even when faced with evidence questioning safety or effectiveness, as well as the ethical implications of therapeutic misconception. Previous research suggests many patients use probiotics because they consider them to be natural or part of complementary and alternative medicine [6]. Additionally, marketing strategies allude to the empowering affects of dietary supplements, such as “be proactive” or “nutrition possible,” and these affects create hope of good health. This political economy of hope depends on simple and quick actions or behaviors to take responsibility for our health, and we feel empowered when we spend the money on dietary supplements that promise good health [32].

Investigators’ primary legal concerns centered on regulation and policy change. Unsubstantiated claims about the effectiveness of these products have the potential to cause harm [33]. Substantive changes at the policy level constitute the most effective way to regulate the current dietary supplements industry, as well as protect the public’s health. Regulations previously advanced under DSHEA [30], including preventing the dietary supplements industry from deceit or manipulation, being truthful about the actual health benefits of their products, requiring manufacturers to list ingredients on each product, and how the product’s dosage is or is not useful (i.e. does the product contain an adequate amount of probiotic to affect positive health?), might be insufficient in the context of the current commercial market. Manufacturers are responsible for ensuring the safety of their products and consumers are responsible for reporting adverse events; the FDA, however, is responsible only for investigating consumer claims that a product is unsafe. Given the increasing availability of over-the-counter dietary supplements, and the profit incentive to produce these supplements, future research should examine the utility of current regulations, specifically whether or not these regulations are in place to protect the public or the market.

There are broader ethical, legal and social implications associated with commercialization and human microbiome research, which should be considered in future research. Sharp and colleagues [7], for example, introduced the environmental impact of probiotics. They raised concerns about negative changes to the microbial ecosystem of the human host, focusing on the different ways in which people process these supplements and the potential for developing immunity to probiotic bacteria. They also discussed concerns about external environmental contamination, focusing on the release of genetically altered or engineered bacteria into the external environment. Future research should consider the long-term impact of internal and external environmental contamination, exploring the ways in which the commercial market has the potential to mitigate or exacerbate these concerns. Additionally, recent research highlights issues of ownership implicated in this type of research. Hawkins and O’Doherty [34] explore the symbiotic relationship between microorganisms and the human body, particularly in the context of private or government biobanks. While their analysis does not focus on issues of commercialization, future research should consider how this issue might play out in a commercial market. The commercialization of human microbiome research, for example, has the potential to give a marketable exchange value to an individual’s microbiome or bacteria. Because the commercial market is focused on profit, there is potential for buying samples to access bacterial sequences for commercial research (e.g. pharmaceutical research).

The purpose of this article was to fill a gap in the literature concerning the commercialization of human microbiome research. Our findings, while not generalizable, suggest that investigators involved in human microbiome research were sensitive to these issues, and were interested in locating effective strategies to inform and protect the public. Future research should explore the ways in which these issues are similarly situated within and across populations currently incorporating dietary supplements. The issues we identified in this study are unlikely to be confined to this particular group of investigators, however through comparative research we can further examine the extent to which these issues exist elsewhere.

## Endnotes

^a^The United States Food and Drug Administration defines dietary supplements as those products taken by mouth in the form of tablets, capsules, or foods, which contain “dietary ingredients,” such as vitamins, minerals, or amino acids (see http://www.fda.gov/Food/DietarySupplements/ConsumerInformation/ucm110417.htm#what).

## Competing interests

The authors declare that they have no competing interests.

## Authors’ contributions

MJS contributed to data collection, carried out the analysis and interpretation of the data presented in this article, and drafted the manuscript. LSA contributed to data collection, analysis, and interpretation of the data presented in the article. SAM and SNW are co-investigators on this project, and contributed to the design of the study, and data collection and analysis. ALM is the principal investigator on this project, and contributed to the study design and coordination, data collection, and data analysis. All authors read an approved the final manuscript.

## Authors’ information

MJS is an Instructor in the Center for Medical Ethics and Health Policy at Baylor College of Medicine. Her primary research interests center on ethical, social, and cultural issues associated with human genomics research. SAM is Associate Professor in the School of Public Health, University of Texas Health Science Center. Her focus on ethical, legal, and social issues in genomics includes working with colleagues on sickle cell disease through a Tanzanian-based consortium of African countries. LSA is a research assistant in the Center for Medical Ethics and Health Policy at Baylor College of Medicine. Her current research focuses on ethical, legal, and social issues in genomics. SNW is a family physician and ethicist at Baylor College of Medicine in Houston, Texas. His primary scholarly interest is in the regulation of biomedical and social/behavioral research with human subjects. ALM is Associate Professor of Medicine and Medical Ethics and Director of the Center for Medical Ethics and Health Policy at Baylor College of Medicine. Her research focuses on legal and ethical issues in genomics.

## Pre-publication history

The pre-publication history for this paper can be accessed here:

http://www.biomedcentral.com/1472-6939/13/28/prepub

## Supplementary Material

Additional file 1 Interview Guide, Investigators and Project Leaders, Ethical, Legal, and Social Dimensions of Human Microbiome Research. Click here for file
