# Supplementary material for: “Snake-oil,” “quack medicine,” and “industrially cultured organisms:” biovalue and the commercialization of human microbiome research
Source: BMC Med Ethics. 2012 Oct 30;13:28. doi: 10.1186/1472-6939-13-28 (PMC3512494; doi:10.1186/1472-6939-13-28)
Supplement: Additional file 1 — Interview Guide, Investigators and Project Leaders, Ethical, Legal, and Social Dimensions of Human Microbiome Research. [file 1472-6939-13-28-S1.pdf]

Interview Guide, Investigators and Project Leaders
The Ethical, Legal, and Social Dimensions of Human Microbiome Research
National Human Genome Research Institute (#R01HG004853)
Amy L. McGuire, JD, PhD Principal Investigator

1. Please briefly tell me about your role with the Human Microbiome Project.

2. What, if any, ethical, legal, or social issues have you encountered while working on the HMP?

3. What, if any, ethical, legal, or social issues have you **not** personally encountered that you think are implicated in human microbiome research?

4. In the future, what will be the major clinical applications of human microbiome research?

> [In other words, in what ways will human microbiome research lead to changes in the way medical professionals treat certain conditions or diseases? How will knowledge of the human microbiome affect the practice of medicine?]

5. What lessons have we learned from other projects that can inform how we deal with any of the issues now posed by microbiome research? Please explain.

6. Of the issues that you've raised, which, if any, are unique to human microbiome research?

> [In other words, which of the issues you identified are not implicated in other areas of genomic research?]

7. Several people we have spoken to have described various benefits and challenges associated with working with a large consortium. Can you give me of an overview of how that works?

8. Is there anything else that we have not asked about related to the ethical, legal, and social implications of human microbiome research that you think we should ask about?

9. Is there anything else you would like to tell us about your experience working on the HMP?
